# Four-dimensional quantitative analysis using FDG-PET in clinical oncology

**DOI:** 10.1007/s11604-023-01411-4

**Published:** 2023-03-22

**Authors:** Nagara Tamaki, Kenji Hirata, Tomoya Kotani, Yoshitomo Nakai, Shigenori Matsushima, Kei Yamada

**Affiliations:** 1 Department of Radiology, Graduate School of Medical Science, Kyoto Prefectural University of Medicine, Kyoto, Japan; 2 Department of Diagnostic Imaging, Hokkaido University Graduate School of Medicine, Sapporo, Japan

**Keywords:** PET, FDG, Quantitative analysis, Cancer, Dynamic whole-body imaging

## Abstract

Positron emission tomography (PET) with F-18 fluorodeoxyglucose (FDG) has been commonly used in many oncological areas. High-resolution PET permits a three-dimensional analysis of FDG distributions on various lesions in vivo, which can be applied for tissue characterization, risk analysis, and treatment monitoring after chemoradiotherapy and immunotherapy. Metabolic changes can be assessed using the tumor absolute FDG uptake as standardized uptake value (SUV) and metabolic tumor volume (MTV). In addition, tumor heterogeneity assessment can potentially estimate tumor aggressiveness and resistance to chemoradiotherapy. Attempts have been made to quantify intratumoral heterogeneity using radiomics. Recent reports have indicated the clinical feasibility of a dynamic FDG PET-computed tomography (CT) in pilot cohort studies of oncological cases. Dynamic imaging permits the assessment of temporal changes in FDG uptake after administration, which is particularly useful for differentiating pathological from physiological uptakes with high diagnostic accuracy. In addition, several new parameters have been introduced for the in vivo quantitative analysis of FDG metabolic processes. Thus, a four-dimensional FDG PET-CT is available for precise tissue characterization of various lesions. This review introduces various new techniques for the quantitative analysis of FDG distribution and glucose metabolism using a four-dimensional FDG analysis with PET-CT. This elegant study reveals the important role of tissue characterization and treatment strategies in oncology.

## Introduction

Various imaging modalities have played important roles in the diagnosis, staging, and therapeutic monitoring of cancer. Positron emission tomography (PET) has recently been applied in several oncological areas. Compared to several other noninvasive imaging modalities, PET is characteristically unique for the in vivo quantitative assessment of tumor characteristics [1, 2]. The accumulation of F-18 fluorodeoxyglucose (FDG) may reflect tumor characteristics based on its metabolic activity, including the membrane glucose transporter protein and hexokinase enzyme. High imaging contrast enhances the detection of these characteristics in many types of cancer [3–5].

Quantitative assessment of FDG uptake can often be used for treatment monitoring after chemotherapy or chemoradiotherapy [6–8]. Numerous studies have considered biochemical changes assessed using FDG-PET as a sensitive marker compared with morphological changes estimated using computed tomography (CT) or magnetic resonance imaging (MRI). In addition, patients with a complete metabolic response after therapy may show better disease-free survival and overall survival than those with any other responses [9–21]. Precise assessment of treatment response is required, mainly because of the rapid progress in new treatments for various cancers.

Whole-body PET imaging allows the accurate staging and restaging of various cancers before and after treatment, in combination with CT or MRI. Such three-dimensional (3-D) analysis of FDG distribution using a high-resolution PET system and appropriate software enables the provision of important quantitative parameters for tissue characterization and treatment strategy. On the other hand, FDG uptake is present in many physiological conditions. Therefore, it is often difficult to differentiate pathological from physiological FDG uptake in routine static PET studies [22–28].

**Fig. 1:**
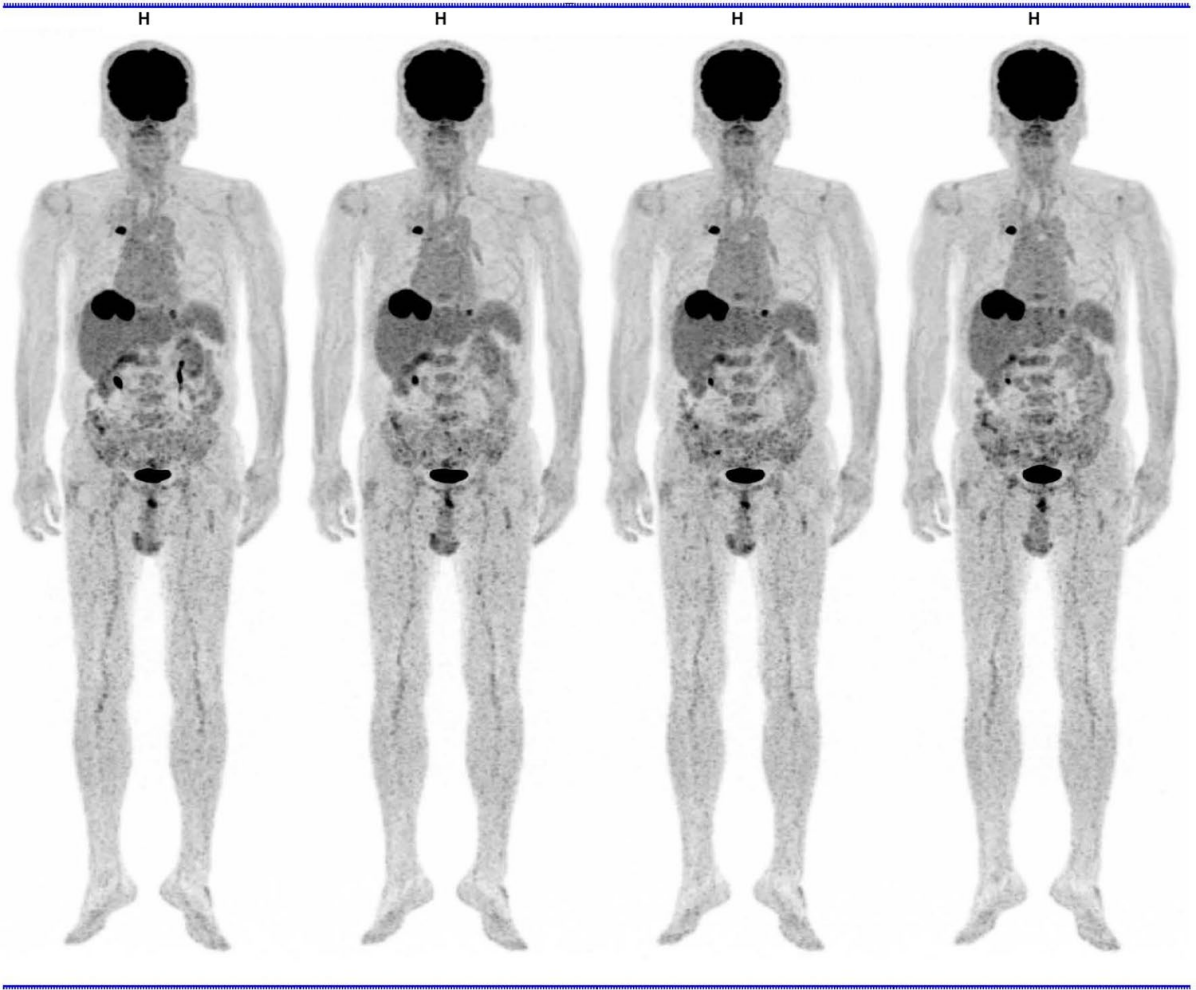
Serial whole-body FDG-PET images (3 min each) approximately 60 min after FDG administration in a patient with lung cancer with pulmonary metastatic lung cancers. High and persistent uptake in the lung cancer and liver metastasis was noted, whereas some uptake motion changes were observed in the ureter and small intestine

With the recent advances in technology, serial dynamic imaging after FDG administration has become possible [29–31]. Such dynamic imaging provides temporal parameters for quantitative analysis of temporal changes in FDG accumulation. Such new imaging methods permit a 3-D spatial analysis of FDG distribution with high-resolution PET imaging, and temporal analysis of FDG uptake changes after administration. Thus, a four-dimensional (4-D) analysis of FDG distribution is now possible and may provide new and valuable information regarding tissue characterization, malignant and benign lesion differentiation, and physiological tracer accumulation [32, 33] **(**Fig. 1).

This review introduces various new techniques for the quantitative analysis of FDG distribution and glucose metabolism with a 4-D FDG analysis using PET-CT. This elegant study highlights the important role of tissue characterization and treatment strategies in oncology.

## Quantitative parameters

### FDG uptake concentration

High-resolution and sensitive PET systems provide several quantitative parameters for FDG distribution and glucose metabolism in oncology. Standardized uptake value (SUV) is one of the most commonly used PET parameters for estimating FDG uptake. The SUV represents the radioactivity concentration in the lesion at a single time point. The SUV is simply the ratio of the activity concentration in the target tissue or lesion to the activity concentration in the whole body. The SUV can be calculated using the following formula: SUV = [tissue tracer activity concentration [Bq/mL]]/ ([injected dose [Bq]]/[body weight [g]]) [6–8]. When the injected tracer was homogeneously distributed throughout the body, the SUV was defined to be 1.

Metabolic changes are often assessed using the SUV value of the tumor. The SUV accounts for the differential partitioning of injected activity within the body. When the same PET camera and scan acquisition parameters are used, reasonable reproducibility of the SUV values can be achieved [34–36]. This SUV is commonly used in clinical oncological studies.

The maximal SUV (SUVmax) of a lesion is independent of the size of the region of interest (ROI). SUVmax is most commonly used in clinical practice because it is simple, reproducible, and readily available using widely used software [5, 34]. SUVmax and not SUVmean was used owing to metabolic heterogeneity or irregular tumor borders. However, the SUVmax is sensitive to image noise and motion. In addition, its value depends on the image quality of the PET/CT system. Notably, the state-of-art PET-CT scanners with high spatial resolution usually produce images with a high SUVmax. Such system-dependent values may cause difficulties in directly comparing various PET scanners’ results.

The peak SUV (SUVpeak) was introduced to overcome the shortcomings of SUVmax as a hybrid of the mean value of radiotracer uptake within the ROI surrounding the highest-intensity voxel (generally 1cm^3^ ROI surrounding the voxel with the highest activity). The SUVpeak is less susceptible to noise and scanner differences in spatial resolution. In addition, an index called SUL has been proposed [34], representing the standardized uptake value using the lean body mass index.

Accordingly, changes in SUVmax within the disease sites were used in one of the first iterations of the PET response criteria [37]. More recently, further refinements in the response criteria have been proposed. In particular, the PERCIST framework [34] has been widely adopted by the nuclear medicine community. Based on the same theoretical constructs as SUV measurement, variations in the analytical method have led to related parameters. These include SUL, representing SUV corrected for lean body mass rather than actual patient weight, and SUVpeak, which is the average of the most intense voxels within a relatively small volume of interest (VOI) [34]. SUVpeak was proposed within the PERCIST criteria and aimed to overcome the potential impact of isolated intense voxels on the results [38].

The patient’s total weight is usually used to normalize the SUV. However, in obese people, SUV is overestimated in lesions and normal tissues because FDG is distributed mainly in non-fatty tissues, whereas the percentage of adipose tissue is high in obese people, with minimal FDG accumulation in the fat. The use of SUL rather than SUV normalization by total weight is recommended for obese patients.

### Volumetric measurement

PET/CT provides an opportunity to evaluate disease burden. The SUVmax metrics indicate the radioactivity concentration of a very small region within the tumor and, therefore, do not consider the tumor volume. At this point, PET differs from CT or MRI, where the tumor size is usually measured using a major axis. Indices for estimating tumor size have been established for PET. Among the various volumetric parameters of FDG PET, metabolic tumor volume (MTV) and total lesion glycolysis (TLG) are commonly used [39]. MTV represents the volume of the tumor with active FDG uptake (which is usually above a certain threshold, such as SUV ≥ 2.5 or SUV ≥ 40% SUVmax). TLG is calculated by multiplying the SUVmean of the total tumor by its MTV. Thus, TLG is the sum of the SUV within a lesion. These volumetric parameters have been used as prognostic indicators for various tumors, as described below.

### Heterogeneity measurement

Many malignant tumors tend to be naturally heterogeneous. Several mechanisms have been used to explain the heterogeneous nature of malignant tumors. First, tumors tend to develop from genetic mutations more frequently than normal cells, leading to heterogeneous biological behavior. Second, tumors grow rapidly, and develop a hypoxic region, resulting in elevated glucose consumption (Warburg effects) or severely decreased metabolism (necrosis). Third, tumor cells coexist with microenvironment cells, such as tumor-associated macrophages (TAM), cancer-associated fibroblasts (CAF), and myeloid-derived suppressor cells (MDSC). The variability of tumor proportions and activities may cause metabolic heterogeneity in cancer tissues as a whole. Generally, tumor heterogeneity may be associated with aggressiveness, growth speed, and metastatic potential, all of which are important for clinical management.

Tumor heterogeneity can be described qualitatively as ‘homogeneous’ or ‘heterogeneous’ (sometimes, ‘inhomogeneous’). In radiology reports of FDG PET-CT, radiologists and nuclear medicine physicians often evaluate tumors using qualitative categories; however, such expressions are subjective and poorly reproducible. Especially, the criteria to distinguish ‘homogeneous’ from ‘heterogeneous’ are unclear. In addition, it is difficult to describe the degree of tumor heterogeneity.

In this context, radiomics has recently been introduced as a technique that uses a mathematical model to quantify heterogeneity by extracting numerical features from radiological images [40]. Individual features only represent image characteristics and are not immediately clinically useful. However, by combining several features and machine learning techniques, such as random forest and support vector machine, some clinical information, such as tumor malignancy, treatment response, and survival, could be predicted.

The simplest radiometric method encloses the tumor and a region of interest (ROI) or volume of interest (VOI) if it is 3-D, creates a histogram of voxel values (usually, SUV) within the ROI/VOI, and calculates the mean, standard deviation, energy, entropy, kurtosis, and skewness. This is typically called a first-order statistic. This may represent tumor heterogeneity to some extent; however, this method does not consider spatial relationships. In other words, this method does not distinguish adjacent from distant voxels. Therefore, when researchers apply radiomics or texture analysis to their data, they usually employ higher-order statistics that can incorporate the spatial distribution of the voxels. To calculate higher-order features, it is necessary to calculate a matrix (that is, an intermediate product) that represents heterogeneity, such as the gray-level co-occurrence matrix (GLCM), gray-level size-zone matrix (GLSZM), gray-level run-length matrix (GLRLM), or neighborhood gray-tone difference matrix (NGTDM). Each matrix generates several features. The imaging biomarker standardization initiative (IBSI) described 174 radiomic features with clear definitions [41]. It may be difficult for beginners in this field to understand this large number of features simultaneously. Thus, here in this article, we would like to focus on the ‘entropy calculated from GLCM’ (EntropyGLCM), which is not only easy to understand but also clinically useful in esophageal and lung cancers [42]. In addition, EntropyGLCM is relatively robust and less sensitive to variability in tumor boundary determination [42].

**Fig. 2:**
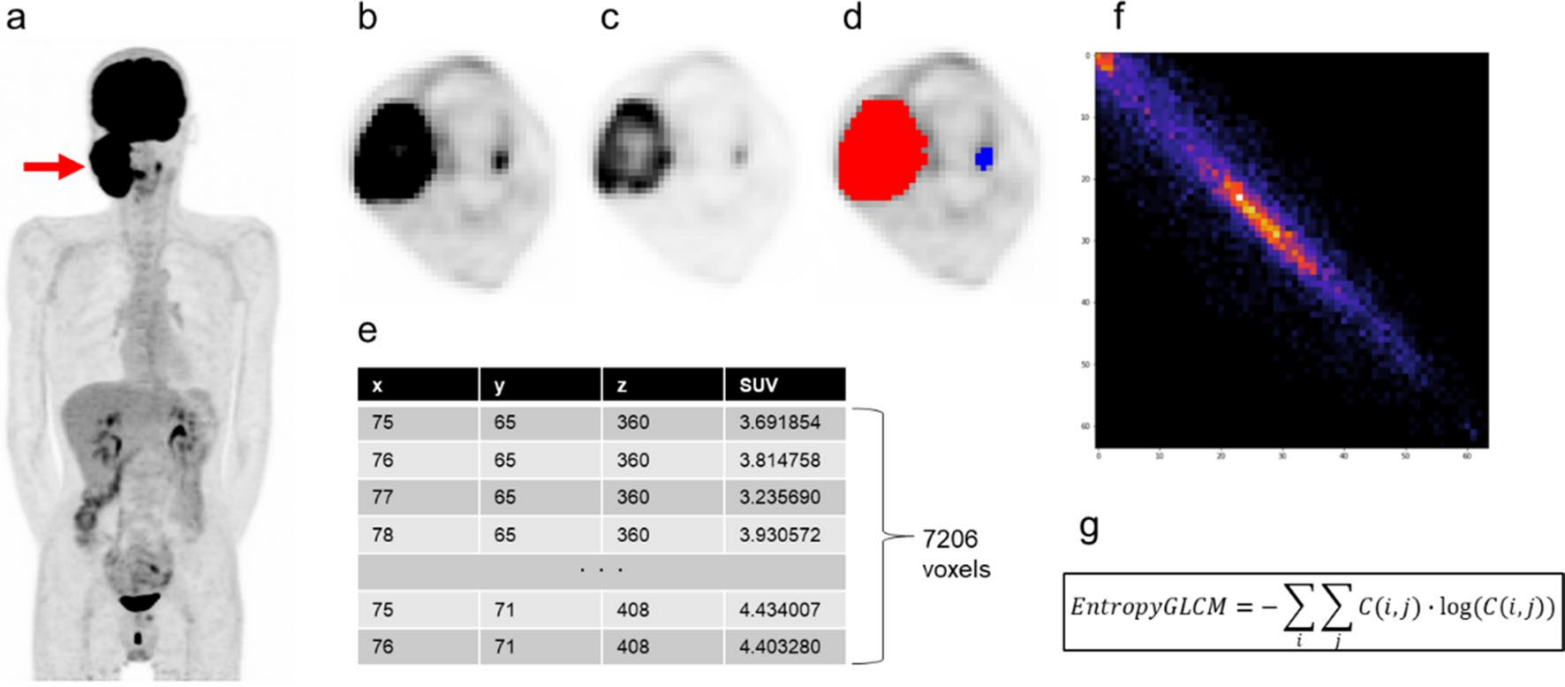
An example of the texture analysis procedure. **a** Maximum intensity projection of FDG-PET of a male patient in his 50 s with diffuse large B-cell lymphoma. A huge tumor in the right neck was shown (arrow). **b** A transaxial slice showing the tumor with SUV window 0 to 6 indicates that the entire tumor metabolizes homogenously; however **c** the same slice with SUV windows 0 to 15 depicts the intra-tumoral heterogeneity of metabolism. **d** The tumor was segmented using SUV≧3 as the criteria. **e** A total of 7206 voxels were extracted from the segmented region. Not only SUV but also the (x,y,z) coordinate are important for the following calculation. **f** After 64-degree discretization, the GLCM (64 × 64) was calculated. **g** EntropyGLCM was calculated from the GLCM. The value was 6.60 in this case

Figure 2 illustrates a case in which EntropyGLCM was calculated from an FDG PET image of a patient with a neck lesion of diffuse large B-cell lymphoma (Fig. 2a, arrow). The first step was to determine the tumor area. If the tumor boundary is determined manually, the reduced reproducibility of VOI reduces the reliability of the texture features. Thus, it is better to segment the tumor automatically. In this case, we delineated the tumor using an SUV of ≥ 3. The red area represents the segmented tumor region (Fig. 2d). All voxels within the region were extracted (Fig. 2e). It is important to record the (*x*, *y*, *z*) coordinates with the SUVs to preserve the locations of the voxels. In this example, 7206 voxels were extracted from the VOI.

The SUV is a continuous value and is often discretized for texture analysis. A common method is to discretize the SUV with a fixed number of bins between the SUVmin and SUVmax (in this example, SUVmin = 3.00, SUVmax = 22.45, and 64 bins). An alternative method is to fix the SUVmin and SUVmax at 0 and 20, respectively.

The GLCM was calculated from the discretized voxel list, which can be regarded as a correlation plot between adjacent voxels. Note that in the 3D space, there are 13 definitions of connected voxel directions whose distance is less than 2 voxels (that is, 1, $\sqrt{2}$ or $\sqrt{3}$); therefore, it is common to compute the GLCM in 13 different ways. For simplification, we focused on voxels adjacent to each other in the x-axis direction, such as (75, 65, 360), and (76, 65, 360). The GLCM is shown as a scatter diagram (Fig. 2f). Note that if all voxel pairs have the same value, the scatter diagram will be the line of identity (*y* = *x*).

Finally, the EntropyGLCM was calculated using the following formula:

$$ {\text{Entropy}}\,GLCM = - \sum \limits_i \sum \limits_j C\left( {i,j} \right) \cdot \log \left( {C\left( {i,j} \right)} \right) $$

where C(i,j) is the GLCM probability value. As it is a ‘probability’ value, C (i, j) is normalized as follows:

$$ \sum \limits_i \sum \limits_j C\left( {i,j} \right) = 1 $$

Similar to the general definition, entropy is the negative of the inner product of the probability and log of the probability values. Entropy can be calculated using either a histogram or GLCM; therefore, it is important to note which one it represents. Different feature names, such as EntropyHist and EntropyGLCM, are commonly used for this purpose.

There are two popular free software tools for radiomics or texture feature calculation: *LIFEx* [43] and *PyRadiomics* [44]. *LIFEx* is an integrated environment with a graphical user interface (GUI). *PyRadiomics* is a python library package. If a GUI is needed for *PyRadiomics*, *3D Slicer* can work together.

Among several problems in radiomics, robustness, and replicability seem to be the largest [45]. Recently, ComBat harmonization was proposed to solve this problem [46, 47].

### Temporal measurement

While most of the FDG parameters are derived through static PET imaging, there are a few trials that assessed temporal changes in FDG distribution using dynamic PET studies. For instance, dual-time-point PET imaging, which obtains both early standard and delayed PET images, has been used to differentiate benign from malignant lesions [31–33]. In addition to these studies that used visual assessment, the retention index (percentage difference in SUV between early and delayed images) was calculated [48].

There are new strategies for the generation of parametric images (pixel-by-pixel analysis) based on graphical analysis, such as the Patlak method [49, 50]. The graphical analysis is simple, and robust, and enables the direct estimation of the primary kinetic macro-components of tracer uptake across multiple fields of view. Serial dynamic whole-body PET imaging is considered a suitable method for assessing temporal changes in tracer uptake. More recently, several reports have indicated the clinical feasibility of dynamic FDG PET-CT in a pilot cohort of oncological cases [51–53].

**Fig. 3:**
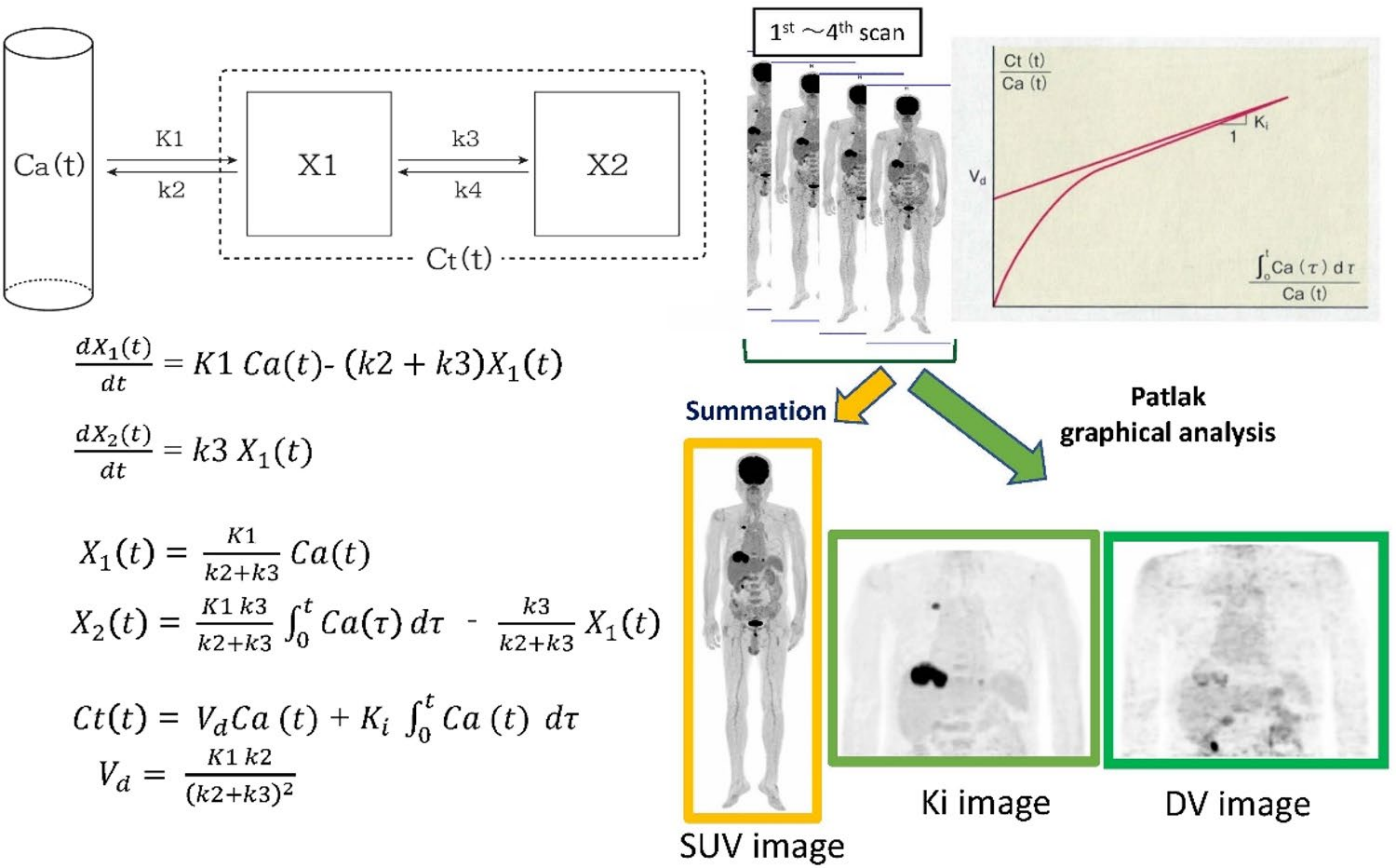
Patlak analysis concept. Ki image and DV image can be obtained from this analysis

The Patlak model can be applied when the tracer reaches a steady state between blood and tissue. This model estimates the Patlak slope (Ki), which is the rate of irreversible uptake, and the Patlak intercept, which is the apparent distribution volume (DV), of the nonmetabolized tracer (Fig. 3). Accordingly, the metabolic rate of FDG was estimated as follows: Metabolic rate of FDG (MRFDG) = Ki × blood glucose [49, 50].

A preliminary study indicated that multi-pass whole-body PET Ki parametric imaging that utilizes robust Patlak graphical analysis may achieve equivalent or potentially, superior lesion detectability than standard-of-care SUV imaging with reduced false-positive rates in routine oncology applications [52]. Ki parametric imaging seems to be particularly valuable for differentiating abnormal lesion uptake with a gradual increase in FDG uptake from physiological uptake areas and a gradual decrease in uptakes, such as in the liver and blood vessels. Ki may reflect count changes; therefore, it has the potential to enhance the detection of abnormal FDG uptake lesions in high background areas [53, 54].

A key issue in the kinetic analysis is obtaining a suitable input function for graphical analysis. Arterial or arterialized venous blood sampling is commonly performed, particularly for brain studies; however, this method is apparently invasive [55–58]. An image-based input function can be obtained using serial dynamic PET imaging, which covers large arterial blood regions such as the left ventricle and aorta [59]. Such a precise FDG kinetic analysis seems rather complicated and remains under investigation. Recently, a standardized input function has been proposed as a surrogate that may facilitate such parametric studies compared to the actual measurement of the input function on rapid dynamic imaging in cardiac areas [60–63]. Serial dynamic FDG-PET imaging using either actual early dynamic imaging or a standardized input function may hold new promise for quantitative analysis of glucose metabolism for pre-vise tissue characterization in a variety of fields, including oncology.

Table 1 summarizes the advantages and disadvantages of each quantitative parameter on 3-D and 4-D PET analyses. While the SUV parameter is commonly used, the 3D PET analysis permits the estimation of the number of volumetric and distribution parameters. New and valuable information regarding metabolic parameters is provided by 4-D analysis. However, such parameters require dynamic acquisition; thus, some difficulties remain in routine clinical settings.

**Table 1:** Various quantitative FDG parameters on 3-D and 4-D PET analysis

Parameters	Estimation	Clinical advantages	Disadvantages
SUV	Uptake concentration (max, peak, or mean)	Well established	Only pixel uptake
MTV	Uptake volume	Prognostic value	Depend on threshold
TLG	Uptake volume × mean uptake	Prognostic value	Depend on threshold
Heterogeneity	Radiomic analysis (texture analysis etc.)	Treatment resistance	Sensitive to scanner performance and analysis method
Ki	Uptake slope (Patlak plot)	Metabolic parameter	Difficult to estimate Dynamic acquisition
DV	Distribution volume (Patlak plot)	?	Difficult to estimate Dynamic acquisition

*SUV* standardized uptake value, *MTV* metabolic tumor volume, *TLG* total lesion glycolysis

## Clinical applications

These quantitative parameters have several clinical applications in clinical oncology. The 3-D quantitative analysis of FDG distribution provides important information for treatment strategies in oncology.

### Risk analysis

Quantitative analyses of FDG uptake have been performed for prognostic studies in patients with lung cancer. The SUV has been used for assessing either the likelihood of malignancy or aggressiveness in lung masses and as a result, the prognosis of known lung cancer has been assessed in many studies [9–17]. For instance, an earlier meta-analysis of 13 such studies evaluated the use of SUV for prognostic stratification of non-small cell lung cancers (NSCLCs) [9]. Several recent reports have suggested SUV analytical values for predicting patient outcomes in lung cancer [10–17]. A similar quantitative analysis of FDG uptake has also been applied in prognostic studies of other cancers [18–21].

Similar to SUV evaluation, volumetric analysis has been used as a prognostic biomarker. Despite the lack of a standardized method for its determination and its significant variability with different thresholds, MTV has a great potential for risk analysis in lung cancer [64, 65]. Moreover, tumor heterogeneity may have the potential for predicting tumor outcomes [66–68]. Heterogenic tumors may contain hypoxic cells that are resistant to chemoradiotherapy [69, 70]. Several PET radiopharmaceuticals are used to identify tumor hypoxia [71–74]. However, FDG, the most commonly used PET radiopharmaceutical, can potentially detect the presence of hypoxia based on heterogeneity distribution.

Radiomics, such as texture analysis, has the potential for semiquantitative analysis of spatial FDG distribution. The heterogeneity of FDG distribution has been used for risk analysis in various cancers [39, 75–77]. Recent reports indicated that the heterogeneity of FDG uptake plays a key role in assessing lesion resistance against various treatments; therefore, the prognostic index uses various radiomics, including texture analysis [39]. Tumor heterogeneity is associated with tumor aggressiveness. Most tumors may have necrotic and hypoxic areas, which may indicate resistance to chemo-radiotherapy. Several recent reports considered such tumor heterogeneity assessed using FDG-PET as a prognostic marker in patients treated with chemoradiotherapy [78–82]. There is no gold standard method for estimating tumor heterogeneity using PET. In addition, suitable chemoradiotherapy has not yet been established for heterogeneous tumors. More clinical studies are needed to establish suitable image analysis and clinical management in these patients.

### Treatment response

As FDG uptake is more sensitive to changes induced by treatments rather than morphological analysis, changes in SUV are considered fundamental components of molecular imaging response criteria. Accordingly, SUV measurements are commonly applied in multicenter trials in oncological treatment studies [34, 37].

The assessment of treatment response using FDG PET-CT plays an important role in optimizing subsequent treatment strategies and predicting patient outcomes. Qualitative evaluation remains the most commonly used approach in clinical practice and quantitative assessment of FDG uptake has been applied for treatment monitoring in most malignancies. Recent reports and review articles evaluated the prognostic values of changes in FDG uptake (SUV change) in various cancers [19, 83–89]. In a recent study involving nine PET/CT scanners across six institutions, quantitative SUV analysis demonstrated the ability of FDG PET-CT to predict 12-month overall survival from immune checkpoint inhibitor therapy using the EORTC, PERCIST, and imPERCIST criteria [90, 91].

Diffuse large B-cell lymphoma (DLBCL) and Hodgkin lymphoma have been the most extensively studied cancers for risk stratification and outcomes using FDG PET/CT. Interim PET performed after two or four cycles of chemotherapy has been proposed as a tool for adapting therapy in patients with good responses [92, 93]. In addition, therapy modification or a more aggressive therapy may be required in patients with progressive disease. High-risk patients are not accurately identified by the current prognostic scoring systems [87]. Qualitative PET analysis using the Deauville criteria, as previously described, has been used for the accurate analysis of treatment effects and outcomes [94, 95]. The prognostic role of quantitative PET parameters, particularly MTV, has been demonstrated in many lymphoma subtypes, including DLBCL [95–98]. Patients with a high disease burden, estimated using the MTV, are at a higher risk of treatment failure and a shorter survival rate.

### Use of temporal change

The assessment of temporal changes in FDG uptake using dynamic imaging has several clinical advantages over static imaging. Serial assessment of changes in FDG uptake, even within a short period, is useful for distinguishing pathological from physiological uptake, especially in the abdominal regions [32, 33, 99]. In the dynamic image evaluation, physiologically changed uptake was frequently found in the gastrointestinal regions and ureters. On the other hand, most benign and malignant lesions showed no visual change in serial images, suggesting the need for a high diagnostic value for differentiating physiological FDG uptake from pathological FDG uptake based on the presence of uptake changes on serial dynamic imaging. These dynamic PET studies may minimize the need for delayed PET imaging [33].

**Fig. 4:**
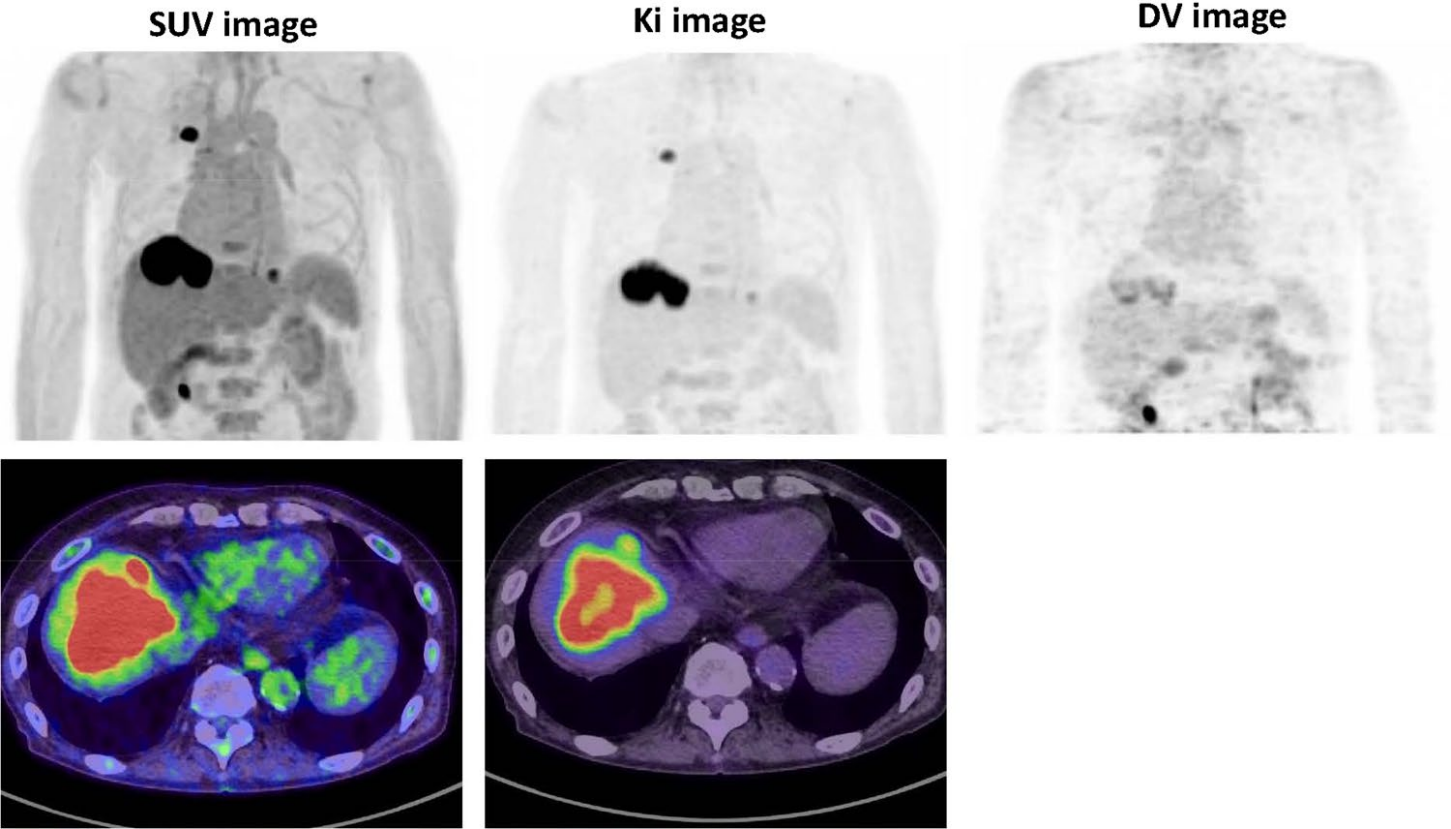
Whole-body (top) images of SUV, Ki, and distribution volume (DV) parameters estimated from the serial dynamic imaging as shown in Fig. 1. Axial images of the SUV and Ki are shown at the bottom. Higher contrast of the lesions was noted with attenuated physiologic uptake in the mediastinum and liver on Ki images

In addition, most malignant lesions may show a gradual increase in FDG uptake, indicating high glucose (or FDG) metabolism, as compared to minimal change or decrease in benign lesions and physiological uptake areas. Recent reports indicated that the target-to-background ratio of Ki in the Patlak analysis was higher than the commonly used SUV values, indicating a higher contrast on the parametric PET images than on the static images [53, 54, 99–101] (Fig. 4). Some of these preliminary studies did not show significant differences in the contrast between malignant and benign lesions. However, Ki, a marker of FDG metabolism, seems to be more suitable for predicting tumor cellular function and aggressiveness than SUV, a static FDG uptake marker in lung and breast cancer [97–105]. As many PET centers have had the chance to apply their up-to-date technology for parametric FDG analysis, these preliminary results will be confirmed in many oncology areas.

PET has significant advantages in the quantitative analysis of tracer activity distributions. In particular, 4-D PET analysis provides new metabolic parameters. A more precise assessment of the clinical values of such parametric imaging is expected in oncology with more patient-based studies in the future.

## Future perspectives

In addition to the introduction of elegant data analyses, instrumental developments have been conducted.

PET-CT systems equipped with silicon photomultiplier–based detectors (digital PET) have been introduced. This new system has improved detection capabilities that might contribute to enhanced diagnostic performance and reduce the activity administration or scan duration. In addition, quantitative capability has been improved significantly owing to a higher spatial resolution and lesser scatter noise [106–108]. Such high performance of the PET system permits short and serial dynamic imaging with high-quality images. Thus, a better quantitative 4-D analysis of FDG PET/CT may be available in clinical oncology [54].

Such digital PET with their magnetic-susceptibility tolerance makes them ideal devices for use in PET-MR systems [109, 110]. There are many advantages of the combined analysis of PET and MRI in the clinical oncology field, which have been fully presented elsewhere [111, 112].

## Conclusion

FDG PET-CT can provide valuable clinical information regarding tumor metabolism and aggressiveness in various types of cancers. Various quantitative approaches have been introduced for image-based precision medicine, including tracer concentration, volume, and heterogeneity analysis using 3-D FDG distribution. Recent progress in PET imaging permits the temporal assessment of FDG uptake. Thus, a 4-D analysis of FDG PET-CT is available for the precise tissue characterization of various lesions. Such elegant studies will provide new and valuable information for tissue characterization and treatment strategies in oncology.
